# Diagnostic Value of the Voltage‐to‐Mass Ratio in Biopsy‐Proven Cardiac Amyloidosis

**DOI:** 10.1111/anec.70026

**Published:** 2024-10-21

**Authors:** Zihan Jiang, Shengsheng Zhuang, Min Tang, Zhuang Tian, Shuyang Zhang

**Affiliations:** ^1^ Arrhythmia Center, State Key Laboratory of Cardiovascular Disease, Fuwai Hospital, National Center for Cardiovascular Diseases Chinese Academy of Medical Sciences Peking Union Medical College Beijing China; ^2^ Department of International Medical Service, Peking Union Medical College Hospital Chinese Academy of Medical Sciences and Peking Union Medical College Beijing China; ^3^ Department of Cardiology The Xiamen Cardiovascular Hospital of Xiamen University Xiamen China; ^4^ Department of Cardiology, Peking Union Medical College Hospital Chinese Academy of Medical Sciences and Peking Union Medical College Beijing China

**Keywords:** amyloid, cardiomyopathy, echocardiography, electrocardiography

## Abstract

**Objectives:**

The calculation of left ventricular mass varies in different studies, and reference values of the voltage‐to‐mass ratio for diagnosing cardiac amyloidosis (CA) are lacking. This study aimed to determine the value of the voltage‐to‐mass ratio in diagnosing CA and provide an optimal cut‐off value for different calculation methods.

**Methods:**

We reviewed the electrocardiograms and echocardiograms of 213 consecutive biopsy‐proven CA patients, 236 hypertrophic cardiomyopathy (HCM) patients, 100 hypertensive heart disease patients, and 181 healthy controls. Left ventricular mass was calculated using linear and cross‐sectional area (CSA) methods. The voltage‐to‐mass ratios were compared between the CA group and other groups. The voltage‐to‐mass ratio obtained was used to build multivariate logistic regression models that predicted the log odds of developing CA.

**Results:**

The CA group had a significantly lower voltage‐to‐mass ratio than the HCM, hypertensive heart disease, and healthy control groups. The voltage‐to‐mass ratio was an independent factor significantly associated with the CA diagnosis after adjusting for baseline characteristics. Linear and CSA methods yielded areas under the ROC curve of 0.86 and 0.90, respectively. Using the CSA method, the optimal cut‐off was 16.42 mV/mm^2^/m^2^, with 89.0% sensitivity and 80.8% specificity.

**Conclusion:**

The voltage‐to‐mass ratio could differentiate patients with CA, HCM, and hypertensive heart disease from healthy controls, potentially providing an accurate and non‐invasive alternative to current expensive and invasive diagnostic techniques.

## Graphical abstract

The cardiac amyloidosis group had a significantly lower voltage‐to‐mass ratio than the HCM, hypertensive heart disease, and healthy control groups, regardless of whether the linear or cross‐sectional area methods were used.

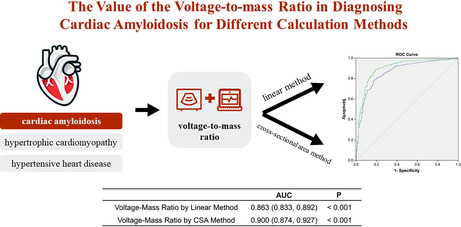

**Abbreviations**
CA: cardiac amyloidosis
CK‐MB: creatine kinase‐MB
CSA: cross‐sectional area
cTnI: Troponin I
DED: dimension at end‐diastole
ECG: electrocardiogram
HCM: hypertrophic cardiomyopathy
IVS: interventricular septum
LVEDD: left ventricular end‐diastolic diameter
LVEF: left ventricular ejection fraction
LVID: left ventricular internal dimension
LVPW: left ventricular posterior wall
MRI: magnetic resonance imaging
NT‐proBNP: N‐terminal‐prohormone B‐type natriuretic peptide
PWT: posterior wall thickness
ROC: receiver operating characteristic
Th: end‐diastolic wall thickness

## 1 Background

Cardiac amyloidosis (CA) is a group of diseases caused by the deposition of amyloid material in the extracellular matrix of the myocardium, resulting in a series of clinical manifestations, such as progressive heart failure and arrhythmias. Some subtypes have a poorer prognosis, namely primary light‐chain CA, which has a median survival of only 6 months after the onset of heart failure symptoms in the natural course of the disease (Kyle et al. 1992). Early diagnosis and treatment are vital to improve prognosis. CA has a high rate of clinical underdiagnosis and misdiagnosis, and is often misdiagnosed as other diseases manifesting as cardiac hypertrophy, such as hypertrophic cardiomyopathy (HCM) or hypertensive heart disease. The gold standard for diagnosing CA is myocardial biopsy (Kapoor et al. 2011), an invasive procedure that increases the risk of arrhythmia, cardiac perforation, and pneumothorax. Among the non‐invasive tests for CA diagnosis, the diagnostic value of cardiac magnetic resonance imaging (MRI) and radionuclide tracer techniques has gradually increased in recent years. However, they can only be performed in a few large medical centers. Therefore, developing more low‐cost and widespread non‐invasive tests is crucial.

Previous studies found that a reduced voltage‐to‐mass ratio may be a characteristic manifestation of CA (Carroll, Gaasch, and McAdam 1982; Simons and Isner 1992). Other diseases that manifest as left ventricular hypertrophy, such as HCM and hypertensive heart disease, have shown normal or significantly increased voltage. However, in previous studies, the sample sizes of previous studies and the formulas for calculating the mass of the left ventricle were not uniform; both linear (Rapezzi et al. 2009) and cross‐sectional area (CSA) (Carroll, Gaasch, and McAdam 1982) methods were used. Additionally, the specific reference value of the voltage‐to‐mass ratio for diagnosing CA has not been calculated. Therefore, in this study, we used patients with HCM, patients with hypertensive heart disease, and healthy individuals (as controls) to explore the value of the voltage‐to‐mass ratio in diagnosing CA and to provide an optimal cut‐off value for different calculation methods.

## 2 Methods

### 2.1 Study Design and Participants

This was a single‐center retrospective study. The study population was divided into a CA group, two disease control groups (HCM and hypertensive heart disease), and a healthy control group. The patient group included patients hospitalized at Peking Union Medical College Hospital between January 1, 2008, and December 31, 2018. The following inclusion criteria were applied: (i) a discharge diagnosis of CA; (ii) biopsy pathology results from our hospital or other hospitals confirming evidence of amyloidosis (biopsy sites including the endocardium, kidney, gingiva, and skin); (iii) initially presented for treatment; and (iv) medical records containing electrocardiogram (ECG) and echocardiogram reports. The exclusion criteria were other comorbidities leading to left ventricular hypertrophy (mitral valve insufficiency, aortic stenosis, and aortic valve insufficiency) and other comorbidities leading to reduced ECG voltage (moderate‐to‐massive pericardial effusion and hypothyroidism). The disease control groups included patients with a discharge diagnosis of HCM or hypertensive heart disease during the same period in the hospital and whose medical records included ECG and echocardiographic findings. Patients with combined acute myocardial infarction and aortic coarctation, those with other comorbidities causing left ventricular hypertrophy (moderate‐to‐severe mitral valve insufficiency, moderate‐to‐severe aortic stenosis, and moderate‐to‐severe aortic valve closure insufficiency), and those with other comorbidities leading to reduced ECG voltage (moderate‐to‐large pericardial effusion and hypothyroidism) were excluded. Healthy controls were randomly selected from healthy volunteers who were examined at our hospital between December 15, 2020, and January 4, 2021. The study protocol conforms to the ethical guidelines of the 1975 Declaration of Helsinki as reflected by the a priori approval from the Ethical Review Board of Peking Union Medical College Hospital, Chinese Academy of Medical Sciences (I‐23PJ040, No. 2022‐PUMCH‐B‐098). Written informed consent was obtained from the patients.

### 2.2 Data Collection

Patient data were collected from electronic medical records, including sociodemographic information (sex and age), height and weight at admission, laboratory tests at admission [creatine kinase‐MB (CK‐MB), Troponin I (cTnI), and N‐terminal‐prohormone B‐type natriuretic peptide (NT‐proBNP)], comorbidities and history of diseases (hypertension, type 2 diabetes, and myocardial infarction), and length of hospital stay. The outcome measures were as follows:

1. ECG: variables included atrial fibrillation, first‐degree AV block, second‐degree AV block, mean p‐R interval, mean QT interval, low voltage of limb lead (amplitude ≤ 0.5 mV in all limb leads), low voltage of chest lead (amplitude ≤ 1.0 mV in all chest leads), and poor R‐wave progression (minimal R‐wave amplitude in leads V1‐V3, or QS type in leads V1 and V2).
2. Echocardiography: variables included (i) structural indices of the heart chambers: anterior–posterior left atrial diameter, septal thickness, end‐diastolic left ventricular internal diameter (LVEDD), end‐systolic left ventricular internal diameter, posterior left ventricular wall thickness, interventricular septum (IVS), ascending aortic root diameter, aortic root diameter, and inferior vena cava width; and (ii) cardiac function indicators: left ventricular shortening fraction, left ventricular ejection fraction (LVEF), tricuspid regurgitation velocity, and mitral E/A. The LV mass was calculated using two methods:
  - The linear method (Lang et al. 2015): LV mass = 0.8 × 1.04 × [(IVS + LVID + PWT)^3^−LVID^3^] + 0.6 and
  - The CSA method (Carroll, Gaasch, and McAdam 1982): CSA = π[(DED/2) + Th]^2^−π(DED/2)^2^ where DED, dimension at end‐diastole; IVS, interventricular septum; LVEDD, left ventricular end‐diastolic diameter; LVID, left ventricular internal dimension; LVPW, left ventricular posterior wall; PWT, posterior wall thickness; Th, end‐diastolic wall thickness. Both methods were individually corrected for the measured LV volumes using the body surface areas of Chinese adults (Hu et al. 1999).
3. Voltage‐to‐mass ratio: It was defined as the ratio of SV1 + RV5 voltage to the corrected LV mass. To facilitate the calculation, the unit of the left ventricular mass was taken as kilograms, and the unit of the left ventricular cross‐sectional area was taken as square millimeters.

The ECGs were imported as scanned image data. Each ECG was interpreted and entered by at least two internal medicine residents; if the entered results were inconsistent (inconsistent results for the categorical variables or ≥ 25% difference in values for the continuous variables), two additional cardiology fellows were asked to review the results independently. If the review results were still inconsistent, a third cardiology attendant was asked to review the results for a second time. Finally, the results of the categorical variables were determined by the majority of the three specialists, and the continuous variables with the most similar values were averaged by the two specialists. The physicians who read the ECGs were unaware of the sample grouping. Echocardiographic data were extracted directly from the medical records, and the LVEF was selected from the numbers collected using the Teichholtz method.

### 2.3 Statistical Methods

Missing variables were excluded from the analysis. For continuous variables, those that met the normal distribution are presented as the mean ± standard deviation, and those that were skewed are presented as the median (quartile). Categorical variables are expressed as percentage composition ratios. For comparisons between groups, a one‐way analysis of variance was used for normally distributed continuous variables. The Kruskal–Wallis nonparametric test was used for skewed continuous variables, and the chi‐square test was used for categorical variables. For comparisons between the two groups, the chi‐square test was performed before the analysis. The Student–Newman–Keuls method was chosen for comparison if the chi‐square value was equal, and Dunnett's T3 test was chosen if the chi‐square value was not equal. Logistic regression analysis was used to evaluate whether the variables were correlated with the diagnosis of CA. Baseline data variables that were significantly different between the groups were selected for inclusion in the regression analysis. To calculate the cut‐off value, a receiver operating characteristic (ROC) curve was plotted with sensitivity as the vertical coordinate and 1‐specificity as the horizontal coordinate, and the optimal cut‐off value was calculated using the Youden index. The data were analyzed using the SPSS statistical software (version 26.0). Differences were considered statistically significant at *p* < 0.05.

## 3 Results

### 3.1 Study Population and Baseline Characteristics

**FIGURE 1:**
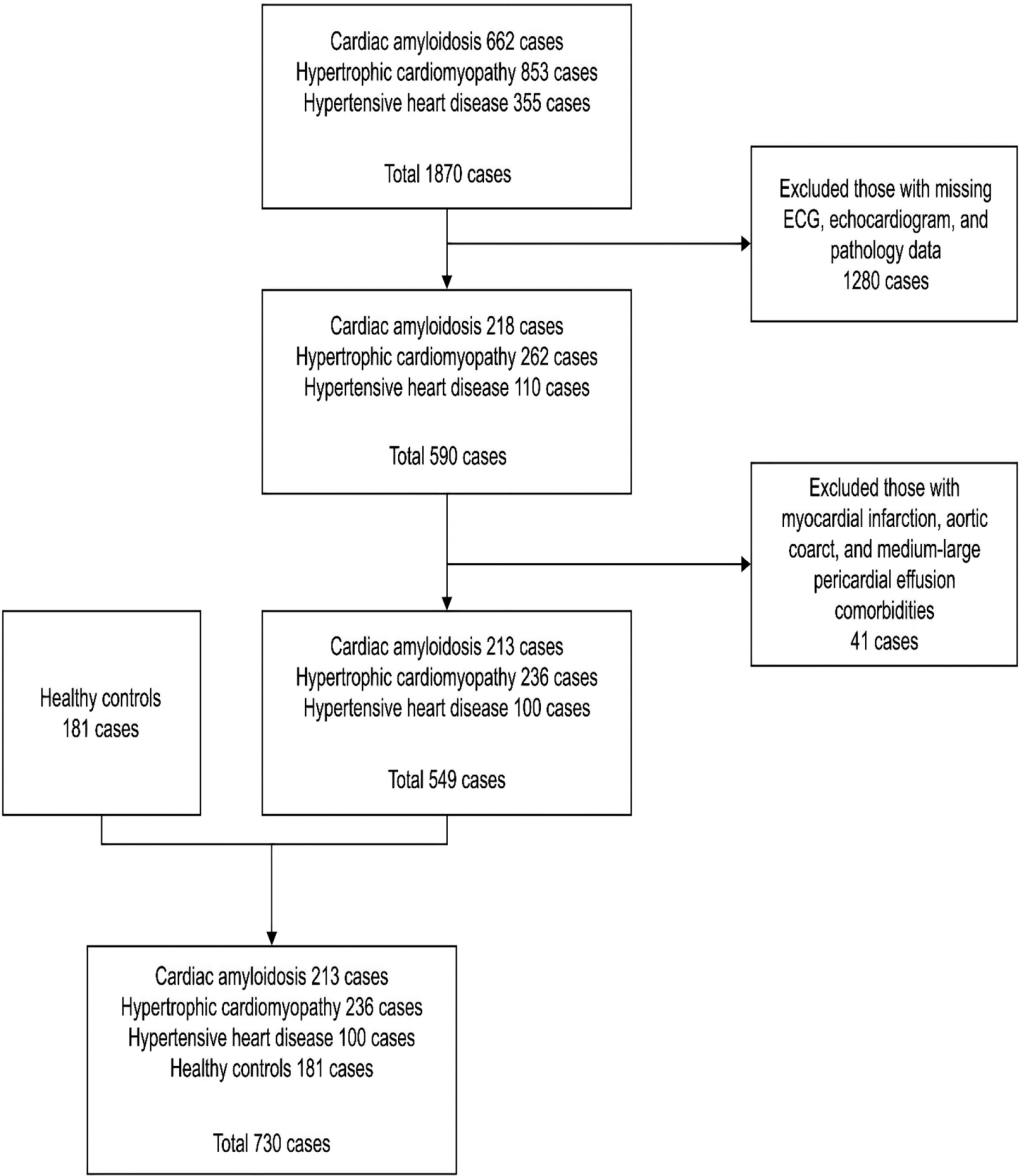
Flowchart regarding the enrollment of the study participants.

Among the patients hospitalized at Peking Union Medical College Hospital between January 1, 2008, and December 31, 2018, 1870 patients met the inclusion criteria, including 662 with CA, 853 with HCM, and 355 with hypertensive heart disease. After excluding patients with incomplete medical records and those with other combined diseases causing extreme abnormalities in the ECGs and echocardiograms, 213 patients with CA, 236 patients with HCM, 100 patients with hypertensive heart disease, and 181 healthy controls were finally included in the study (Figure 1).

The mean age at admission was 58 (49–64) years in the CA group, 61.5 (53–70) years in the HCM group, 47 (32–62) years in the hypertensive heart disease group, and 48 (43–54) years in the healthy control group. In the CA group, 62.4% of the patients were male, and there was no significant difference in sex between the groups. Compared to the other two disease groups, the prevalence of previous hypertension was significantly lower in the CA group (13.1% vs. 50.0% and 100.0%, respectively; *p* < 0.001), as was the prevalence of diabetes (5.6% vs. 19.7% and 23.0%, respectively; *p* < 0.001). The CK‐MB, cTnI, and NT‐proBNP levels at admission were significantly higher in the CA group than in the other two disease groups (Table 1).

**TABLE 1:** Baseline data regarding the patients at admission.

	Cardiac amyloidosis (*n* = 213)		Hypertrophic cardiomyopathy (*n* = 236)		Hypertensive heart disease (*n* = 100)		Healthy controls (*n* = 181)		*p*
	Results	Effective sample size	Results	Effective sample size	Results	Effective sample size	Results	Effective sample size	
Age (years)	58 (49, 64)	213	62 (53, 70)	236	47 (32, 62)	100	48 (43, 54)	181	< 0.001
Male sex (%)	62.4	213	57.2	236	69.0	100	61.9	181	0.23
Body surface area (m^2^)	1.81 (1.66, 1.91)	213	1.88 (1.70, 2.02)	236	1.94 (1.76, 2.16)	100	1.90 (1.75, 2.03)	181	< 0.001
History of hypertension (%)	13.1	213	50	236	100	100	0	181	< 0.001
History of diabetes (%)	5.6	213	19.9	236	23.0	100	0	181	< 0.001
History of myocardial infarction (%)	0	213	3	236	0	100	0	181	0.002
Length of stay (days)	15 (9, 25)	213	15 (8, 27)	236	18 (13, 27)	100	0	181	0.109
CK‐MB level on admission (μg/L)	2.30 (1.50, 3.60)	167	1.60 (0.95, 3.15)	213	1.25 (0.60, 2.40)	88	/		< 0.001
cTnI level on admission (μg/L)	0.11 (0.04, 0.24)	185	0.04 (0.02, 0.14)	217	0.03 (0.01, 0.08)	92	/		< 0.001
NT‐proBNP level on admission (pg/mL)	4917.00 (2626.00, 9579.00)	167	1264.00 (480.00, 3138.00)	155	2697.00 (532.50, 12,065.50)	65	/		< 0.001

Abbreviations: CK‐MB, creatine kinase‐MB; cTnI, Troponin I; NT‐proBNP, N‐terminal‐prohormone B‐type natriuretic peptide.

### 3.2 Echocardiographic Findings

Regarding the echocardiographic findings, the mean values of the anteroposterior diameter of the left atrium were higher than normal in all three disease groups, suggesting that left atrial enlargement is a common manifestation of these three cardiomyopathies. However, the degree of left atrial enlargement was significantly lower in the CA group than in the other two disease groups. Meanwhile, the mean values of septal thickness were higher than normal in all three disease groups: 13.53 ± 3.22 mm in the CA group, 16.00 ± 4.71 mm in the HCM group, and 11.99 ± 2.56 mm in the hypertensive heart disease group. Septal thickening was more common in the HCM group than in the other two disease groups; however, left ventricular posterior wall thickening was the least common in all three disease groups. The LVEF was normal in all three disease groups. Regarding the left ventricular mass, when assessed using the linear method, the left ventricular mass in the CA group was significantly lower than that in the other two disease groups, with 123.56 ± 39.83 g, 129.89 ± 49.00 g, and 137.68 ± 42.32 g in the CA, HCM, and hypertensive heart disease groups, respectively. However, when the cross‐sectional method was used, the left ventricular mass in the HCM group was the lowest among the three disease groups (Table 2).

**TABLE 2:** Results of the echocardiogram, electrocardiogram, and voltage‐to‐mass ratio.

	Cardiac amyloidosis (*n* = 213)		Hypertrophic cardiomyopathy (*n* = 236)		Hypertensive heart disease (*n* = 100)		Healthy controls (*n* = 181)		*p*
	Results	Effective sample size	Results	Effective sample size	Results	Effective sample size	Results	Effective sample size	
Echocardiogram									
Ascending aorta diameter (mm)	32.32 ± 4.13	212	34.74 ± 4.32*	233	35.09 ± 4.73*	99	31.21 ± 3.63*	181	< 0.001
Inner diameter of the aortic root (mm)	31.92 ± 3.52	213	33.20 ± 4.00*	235	33.27 ± 3.87*	100	31.25 ± 3.25	181	< 0.001
Anteroposterior diameter of the left atrium (mm)	41.02 ± 5.96	213	42.86 ± 7.20*	236	43.51 ± 6.44*	100	33.85 ± 3.91*	181	< 0.001
Ventricular septal thickness (mm)	13.53 ± 3.22	213	16.00 ± 4.71*	236	11.99 ± 2.56*	100	7.83 ± 1.23*	181	< 0.001
Left ventricular end‐diastolic inner diameter (mm)	43.46 ± 5.83	213	46.65 ± 5.77*	236	55.28 ± 8.31*	100	46.33 ± 3.41*	181	< 0.001
Left ventricular end‐systolic diameter (mm)	30.63 ± 6.19	213	29.09 ± 5.91	235	39.60 ± 10.10*	100	29.23 ± 2.90	181	< 0.001
Left ventricular fractional shortening (%)	29.74 ± 8.77	213	37.93 ± 7.62*	235	29.20 ± 9.54	100	36.97 ± 3.79*	181	< 0.001
Left ventricular posterior wall thickness (mm)	12.91 ± 2.74	213	10.11 ± 3.00*	236	11.72 ± 2.30*	100	7.61 ± 1.07*	181	< 0.001
Left ventricular ejection fraction (%)	56.00 ± 13.12	213	66.98 ± 10.52*	235	53.25 ± 14.99*	99	66.66 ± 5.02*	181	< 0.001
Tricuspid regurgitation velocity (cm/s)	2.59 ± 0.47	211	2.44 ± 0.50*	220	2.56 ± 0.58	97	2.21 ± 0.28*	181	< 0.001
Mitral valve E/A	1.72 ± 1.00	165	0.89 ± 0.54*	167	1.22 ± 0.67*	70	1.20 ± 0.29*	180	< 0.001
Inferior vena cava width (mm)	16.68 ± 3.33	206	14.95 ± 3.20*	215	15.77 ± 3.29*	95	13.88 ± 2.09*	181	< 0.001
Left ventricular mass by the linear method (g/m^2^)	123.56 ± 39.83	213	129.89 ± 49.00	236	137.68 ± 42.32*	100	60.84 ± 13.46*	181	< 0.001
Left ventricular mass by the CSA method (cm^2^/m^2^)	12.88 ± 3.29	213	9.77 ± 3.62*	236	12.46 ± 3.08	100	6.84 ± 1.14*	181	< 0.001
Electrocardiogram									
Sinus rhythm (%)	84.5	213	75.8	236	89.9	100	100	181	< 0.001
Atrial fibrillation (%)	12.2	213	19.1	236	10.1	100	0	181	< 0.001
Average p‐R interval (ms)	161.01 ± 62.20	204	128.54 ± 71.28*	225	151.87 ± 51.77	100	162.03 ± 20.52	181	< 0.001
First‐degree atrioventricular block (%)	17.8	213	7.2	236	10.1	100	0.6	181	< 0.001
Second‐degree atrioventricular block (%)	0.5	213	0	236	0	100	0	181	0.489
Low voltage of limb leads (%)	50.2	213	5.1	236	2	100	1.7	181	< 0.001
Low voltage of chest lead (%)	6.6	213	3.4	236	0	100	0	181	< 0.001
Poor R wave progression (%)	56.8	213	8.9	235	11.1	100	0	181	< 0.001
Pseudoinfarction pattern (%)	34.7	213	5.1	234	3	100	0.6	181	< 0.001
Average QTc interval (ms)	459.03 ± 41.97	213	464.71 ± 41.05	235	453.5 ± 37.22	100	417.02 ± 30.01*	181	< 0.001
Sv1 + rv5 voltage (mV)	1.44 ± 0.87	213	2.93 ± 1.33*	234	3.43 ± 1.32*	100	1.91 ± 0.54*	181	< 0.001
Voltage‐to‐mass ratio by the linear method (mv/kg/m^2^)	12.49 ± 8.29	213	24.35 ± 12.90*	236	26.51 ± 12.39*	100	32.80 ± 11.49*	181	< 0.001
Voltage‐to‐mass ratio by the CSA method (mv/mm^2^/m^2^)	11.96 ± 8.30	213	31.73 ± 15.63*	236	28.91 ± 14.58*	100	28.66 ± 9.28*	181	< 0.001

Abbreviation: CSA, cross‐sectional area.

* Significant difference compared to the cardiac amyloidosis group alone (*p* < 0.05).

### 3.3 Electrocardiographic Findings

Regarding the electrocardiographic results, the probabilities of a first‐degree AV block, low voltage in the limb and chest leads, poor R‐wave progression, and pseudo‐infarction pattern were significantly higher in the CA group than in the other two disease groups (Table 2). Regarding the voltage‐to‐mass ratio, the values were significantly lower in the CA group than in the other disease groups and healthy control group, using either the linear or CSA methods.

### 3.4 Voltage‐to‐Mass Ratio as an Independent Factor Suggesting CA

The regression analysis was performed to clarify the significance of the voltage‐to‐mass ratio in identifying CA. First, the healthy controls were temporarily excluded, and patients with HCM and hypertensive heart disease were combined into one group and compared with the CA group. Age, sex, laboratory test results reflecting disease severity at admission (CK‐MB, cTnI, and NT‐proBNP), and the voltage‐to‐mass ratio were included in the regression analysis. The results showed that a reduced voltage‐to‐mass ratio was an independent factor suggesting CA, using either the linear or CSA methods. The odds ratios were 1.140 (1.104, 1.178) for the linear method and 1.165 (1.127, 1.205) for the CSA method (*p* < 0.001). The healthy controls were also included in the regression analysis. Patients with HCM, those with hypertensive heart disease, and healthy controls were combined into the “non‐CA group.” The common baseline variables of age and sex were included in the regression analysis. The results showed that a reduced voltage‐to‐mass ratio remained an independent factor suggesting CA, using either the linear or CSA methods (see Figures S1 and S2 in File S1).

### 3.5 The ROC Curve and Optimal Cut‐Off Value for the Voltage‐to‐Mass Ratio

**FIGURE 2:**
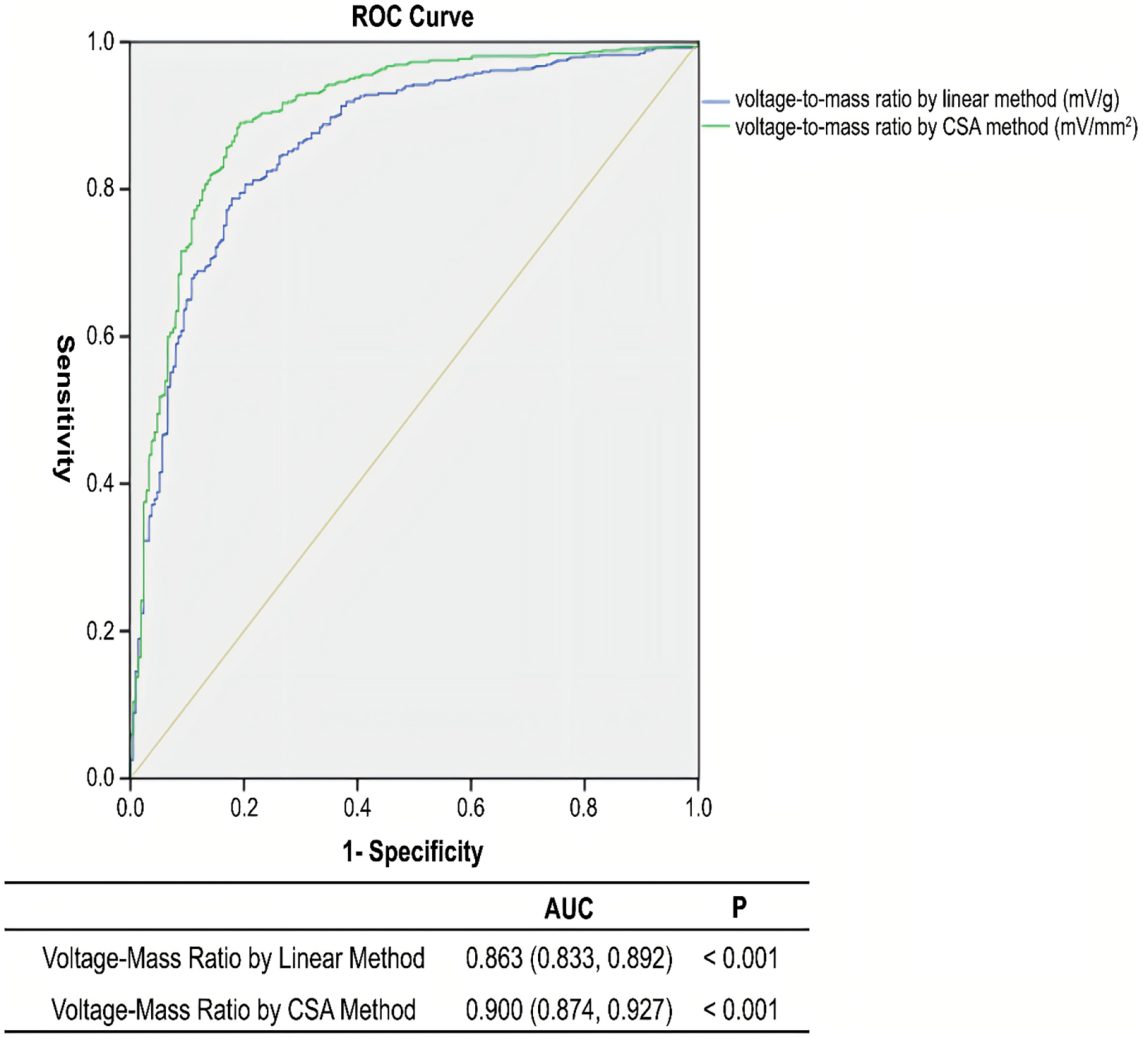
The receiver operating characteristic (ROC) curve and area under the curve (AUC) for the diagnosis of cardiac amyloidosis based on the voltage‐to‐mass ratio. CSA, cross‐sectional area.

Finally, 517 patients with HCM, hypertensive heart disease, and healthy controls were combined into a “non‐CA group.” The ROC curve and maximum area under the curve (AUC) were used to derive the optimal cut‐off value for the voltage‐to‐mass ratio diagnosis of CA (Figure 2). The AUC for the linear method of the voltage‐to‐mass ratio was 0.863 (0.833, 0.892), and that for the CSA method was 0.900 (0.874, 0.927). The Youden index was used to select the cut‐off value. For the linear method, when the voltage‐to‐mass ratio was 17.98 (mV/kg/m^2^), the Youden index for the diagnosis of CA was the highest, with a sensitivity of 78.7% and a specificity of 82.2%. For the CSA method, when the voltage‐to‐mass ratio was 16.42 (mV/mm^2^/m^2^), the Youden index for the diagnosis of CA was the highest, with a sensitivity of 89.0% and a specificity of 80.8%.

## 4 Discussion

Cardiac amyloidosis is a group of cardiac diseases with high rates of late mortality and has a severe impact on patients' quality of life. CA has a high rate of underdiagnosis and misdiagnosis, and is often misdiagnosed as HCM or hypertensive heart disease, which results in delayed treatment. The gold standard for the diagnosis of CA is myocardial biopsy, which is associated with a high risk for patients. Myocardial MRI or radionuclide examination is costly, and neither of these diagnostic modalities can be easily performed in primary care institutions. This study confirms that the combined use of echocardiography and electrocardiography, two non‐invasive and inexpensive tests, is of great value in the diagnosis of CA. Regardless of whether the linear or CSA method was used, the voltage‐to‐mass ratio was a good index for diagnosing CA, and the diagnostic effect of the CSA method was slightly better than that of the linear method. The recommended cut‐off value of the voltage‐to‐mass ratio calculated using the CSA method was 16.42 (mV/mm^2^/m^2^). When the CSA method was used, the sensitivity and specificity for diagnosing CA were 89.0% and 80.8%, respectively.

Echocardiography is essential for diagnosing CA. The characteristic echocardiographic changes in CA are left ventricular hypertrophy without left ventricular dilatation and abnormal diastolic function at an early stage (Tuzovic et al. 2017; Falk et al. 2016). In the present study, the left ventricular end‐diastolic internal diameter was significantly lower in the CA group than in the other two disease groups and the healthy control group. Moreover, the mitral E/A ratio was significantly higher in the CA group than in the other two disease groups and healthy control group and approached severe diastolic limitations with a mitral E/A of 1.72 ± 1.00. This result is consistent with those of previous studies. In the differentiation of CA, HCM, and hypertensive heart disease, although morphologically, all three diseases exhibited left ventricular hypertrophy, patients with CA and hypertensive heart disease showed homogeneous thickening of the left ventricle. In contrast, HCM tends to cause asymmetric hypertrophy, in which the septum is significantly thicker than the posterior wall of the left ventricle, with a corresponding increase in left ventricular systolic function (Mörner et al. 2005). The results of our study are also consistent with these findings. When estimating the LV mass, the linear method included three variables: septal thickness, LV posterior wall thickness, and LV end‐diastolic internal diameter; however, the CSA method included only two variables: LV posterior wall thickness and LV end‐diastolic internal diameter. Because HCM exhibits asymmetric hypertrophy with predominant septal thickening, the LV mass was significantly greater in the CA group than in the HCM group when the CSA method was used. However, no significant difference was observed between the two groups when the linear method was used.

Concerning ECGs, previous studies have shown that poor R‐wave progression, QTc prolongation, and pseudo‐infarction patterns are the most common ECG abnormalities in patients with CA (Cyrille et al. 2014; Murtagh et al. 2005; Rahman et al. 2004). Our study suggested that poor R‐wave progression (56.8%), low voltage in the limb leads (50.2%), and pseudo‐infarction patterns (34.7%) were the most common ECG abnormalities in the CA group. Further, the probabilities of these abnormalities were higher than those in the HCM and hypertensive heart disease groups. Therefore, when echocardiography indicates homogeneous left ventricular hypertrophy, the presence of ECG abnormalities is more likely to indicate CA. The pathophysiological mechanisms underlying poor R‐wave progression in the thoracic leads of patients with CA may be related to myocardial ischemia and impaired wall coordination owing to left ventricular hypertrophy, which is also a possible mechanism for the generation of pseudo‐infarction‐type waveforms (DePace et al. 1983).

The pathophysiological mechanisms underlying low voltage in the limb leads in patients with CA are not fully understood. They may be related to ischemic cardiomyocyte damage, which results in an insufficient number of cardiomyocytes to generate and transmit electrical signals. In addition, a comparative study between ATTR‐type and AL‐type CA suggested that the occurrence of low voltage in the limb leads may also be related to the type of mutation; patients with V122I and Thr60Ala mutations more frequently present with low voltage in the limb leads (Cyrille et al. 2014).

A reduced voltage‐to‐mass ratio is considered to be a characteristic manifestation of CA. When diagnosing CA, using a reduced voltage‐to‐mass ratio is more sensitive than using electrocardiography and echocardiography alone (Simons and Isner 1992). The regression analysis results in this study suggest that a reduced voltage‐to‐mass ratio is an independent indicator suggestive of a diagnosis of CA, regardless of whether a healthy control population was included. Furthermore, by comparing 213 patients with CA with 517 patients without CA, the area under the ROC curve was found to be > 85% for both the linear and CSA methods for calculating the voltage‐to‐mass ratio, further indicating that the voltage‐to‐mass ratio has a good diagnostic value for the diagnosis of CA. When choosing between the two methods for calculating the voltage‐to‐mass ratio, this study suggested that the AUC of the CSA method was slightly better than that of the linear method, indicating that the diagnostic value of the CSA method for calculating the voltage‐to‐mass ratio was better for the samples in our study. Regarding the cut‐off value, the linear method had a sensitivity of 78.7% and a specificity of 82.2% for diagnosing CA at a cut‐off value of 17.98 (mV/kg/m^2^). In comparison, the CSA method had a sensitivity of 89.0% and specificity of 80.8% at a cut‐off value of 16.42 mV/mm^2^/m^2^. The sensitivity and specificity of both methods were good, and both could be used as important non‐invasive screening indicators for the clinical diagnosis of CA.

### 4.1 Study Limitations

The present study has some limitations. First, it was a single‐center study. Thus, whether the cut‐off values and their sensitivities and specificities apply to other centers require further confirmation in a multicenter study. Second, because this was a retrospective study, the enrolled cases spanned a large period, and the echocardiographic results were extracted from medical records rather than from the original image data interpreted by the same cardiac ultrasound expert. Therefore, there may have been subtle artificial measurement differences in the echocardiographic results. Third, the present study did not classify the CA subtypes. Whether there is a difference in the ECG and echocardiographic presentation of different CA subtypes has also been inconsistent in previous studies (Rapezzi et al. 2009; Cyrille et al. 2014). Therefore, the effects of different amyloidosis subtypes on the voltage‐to‐mass ratio require further investigation.

### 4.2 Conclusion

In this study, we analyzed 213 patients with CA confirmed by pathological biopsy and compared them to 236 patients with HCM, 100 patients with hypertensive heart disease, and 181 healthy adults to confirm the clinical value of the combined application of echocardiography and electrocardiography for the diagnosis of CA. Both the linear and CSA methods for calculating the voltage‐to‐mass ratio have good sensitivity and specificity for diagnosing CA.

## Author Contributions

Z.J. and S.Z. have made substantial contributions to the conception, design of the work; acquisition and analysis of data; and have drafted the work or substantively revised it. M.T. has revised the work. Z.T. and S.Z. have made substantial contributions to the conception, design of the work, and revised the work.

## Ethics Statement

The study protocol received approval from the Ethical Review Board of Peking Union Medical College Hospital, Chinese Academy of Medical Sciences (I‐23PJ040, No. 2022‐PUMCH‐B‐098). Written informed consent was obtained from the patients.

## Consent

The authors have nothing to report.

## Conflicts of Interest

The authors declare no conflicts of interest.

## Supporting information


**Figure S1** The effect of the voltage‐to‐mass ratio when the cardiac amyloidosis group was compared with hypertrophic cardiomyopathy, hypertensive heart disease, and healthy control groups.
**Figure S2** The effect of the voltage‐to‐mass ratio when the cardiac amyloidosis group was compared with the hypertrophic cardiomyopathy and hypertensive heart disease groups.

## Data Availability

The deidentified participant data will not be shared.
