# Supplementary material for: Diagnostic Value of the Voltage‐to‐Mass Ratio in Biopsy‐Proven Cardiac Amyloidosis
Source: Ann Noninvasive Electrocardiol. 2024 Oct 21;29(6):e70026. doi: 10.1111/anec.70026 (PMC11492296; doi:10.1111/anec.70026)
Supplement: Supplementary file 1 — Figure S1 The effect of the voltage‐to‐mass ratio when the cardiac amyloidosis group was compared with hypertrophic cardiomyopathy, hypertensive heart disease, and healthy control groups. Figure S2 The effect of the voltage‐to‐mass ratio when the cardiac amyloidosis group was compared with the hypertrophic cardiomyopathy and hypertensive heart disease groups. [file ANEC-29-e70026-s001.docx]

**Additional file 1**


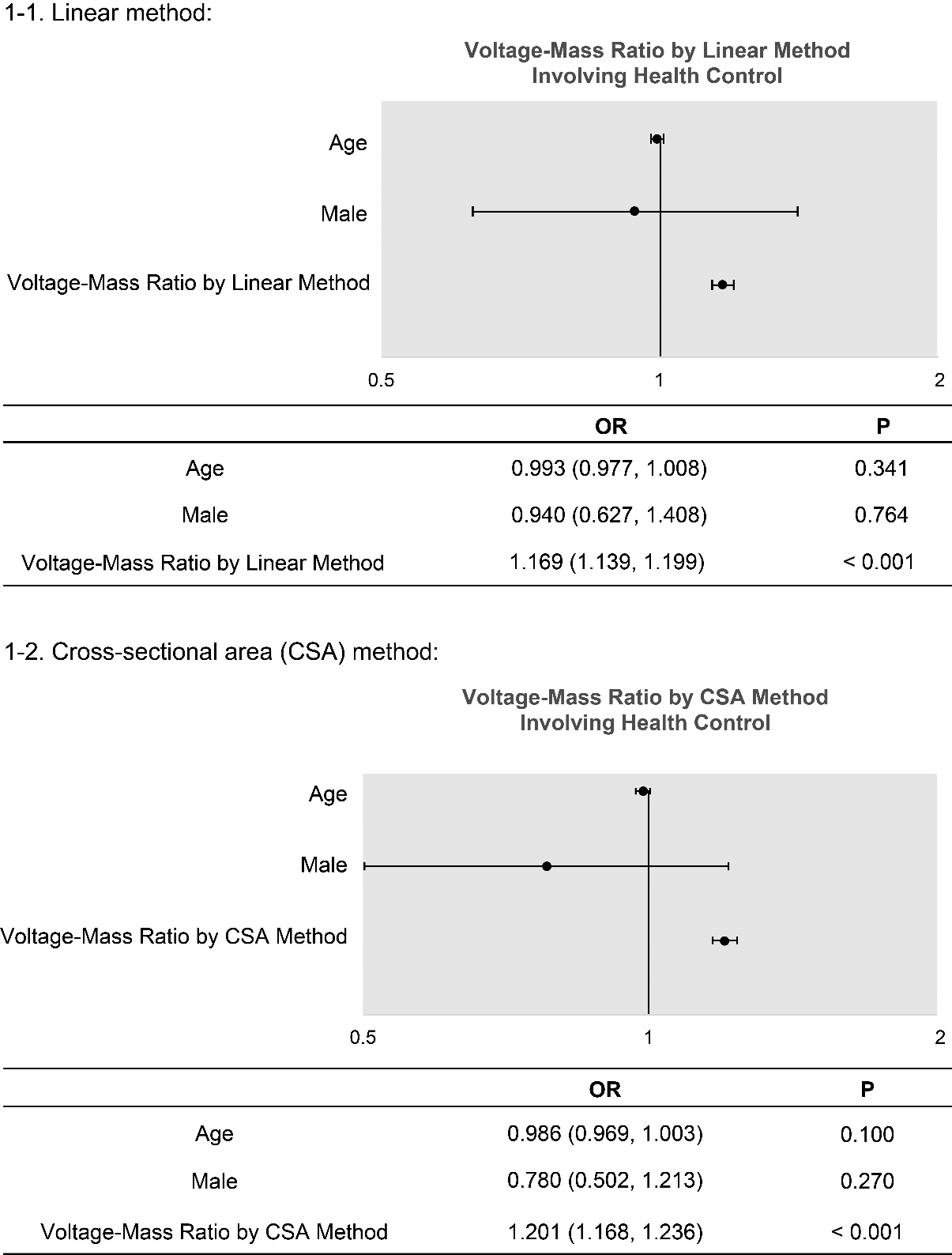


**Figure S1**. The effect of the voltage-to-mass ratio when the cardiac amyloidosis group was compared with hypertrophic cardiomyopathy, hypertensive heart disease, and healthy controls groups. (1-1. Linear method, 1-2. Cross-sectional area (CSA) method.) OR, odds ratio

2-1. Linear method


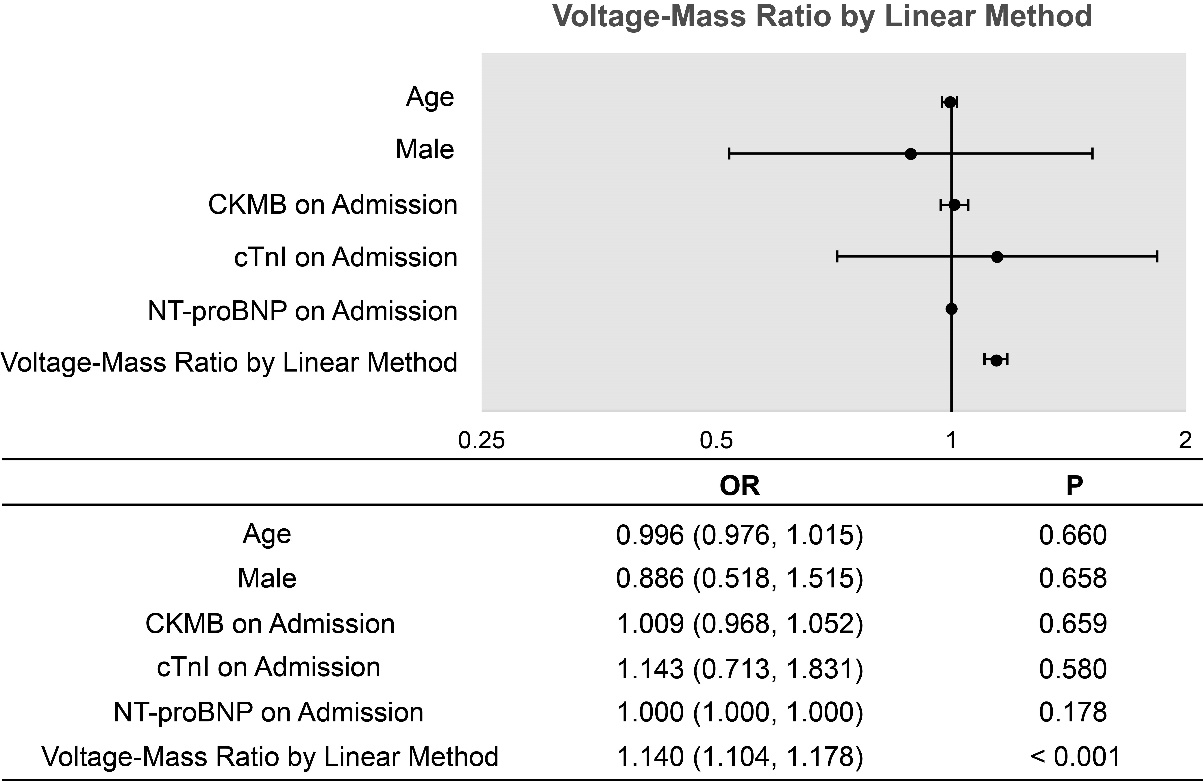


2-2. Cross-sectional area (CSA) method


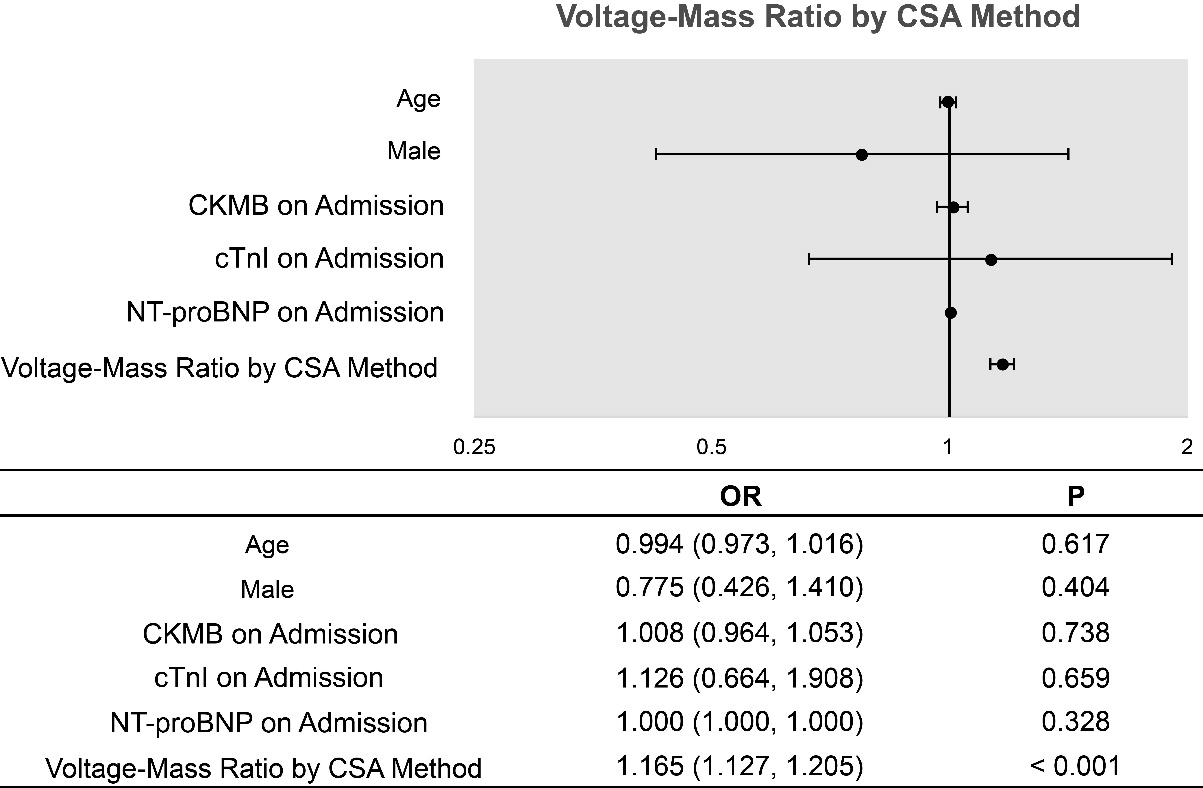


**Figure S2.** The effect of the voltage-to-mass ratio when the cardiac amyloidosis group was compared with the hypertrophic cardiomyopathy and hypertensive heart disease groups (2-1. Linear method, 2-2. Cross-sectional area (CSA) method.) CK-MB, creatine kinase-MB; cTnI, Troponin I; NT-proBNP, N-terminal -pro hormone B-type natriuretic peptide; OR, odds ratio
